# The relevance of digital mental healthcare during COVID-19: Need for innovations

**DOI:** 10.3126/nje.v10i4.32519

**Published:** 2020-12-31

**Authors:** Sujita Kumar Kar, Shailendra K Saxena, Russell Kabir

**Affiliations:** 1 Department of Psychiatry, King George's Medical University, Lucknow, India; 2 Centre for Advanced Research (CFAR), Faculty of Medicine; King George’s Medical University, Lucknow, India; 3 School of Allied Health, Faculty of Health, Education, Medicine, and Social Care, Anglia Ruskin University, Chelmsford, United Kingdom

Sir,

The emergence of the COVID-19 pandemic and its rapid spread globally compromised healthcare delivery globally, including India. Digital health services are adopted worldwide to meet the healthcare needs of the people [1]. Digital platforms started providing online consultation for various medical conditions and mental illnesses [2]. As the digital platform is new for most patients and healthcare providers in India and ethical dilemmas were present with this modality of consultation, there was a reluctance to adopt it initially 3. However, the Government of India has issued guidelines for teleconsultation and promoted its use, which resulted in increasing acceptance of teleconsultation [1].

Tele-consultation for mental illnesses is running in India over the past two decades [4]. Diagnoses of most mental illnesses are based on detailed clinical history and interview of the patient (mental status examination). Physical examination, though an essential part of clinical evaluation, its role is less for making psychiatric diagnosis, unlike its essential role in various other medical disciplines. Hence, teleconsultation in mental health can suffice for the diagnosis of mental illnesses and psychotherapy and prescribing medications. In developing countries like India, the treatment gap for mental illnesses is approximately 85% [5]. Due to several reasons (social stigma, unawareness, financial crisis), people with mental illness cannot reach the mental health facilities for treatment.

COVID-19 may be considered an opportunity to enrol these deprived people in digital healthcare by creating awareness and providing digital healthcare facilities in remote areas by improving internet service. Teleconsultation can provide cost-effective service at the doorstep with adequate client satisfaction [6] at the same time it facilitate early enrolment of patient in clinical care by reducing the duration of untreated illness [7]. Teleconsultations have been effectively used for various groups of patients (e.g: children & adolescents, elderly, psychotherapy, addiction management) with diverse healthcare needs. Early evidences suggest that teleconsultation may serve as a cost-effective, convenient, feasible and effective modality to provide consultation to patient in the follow up care [8]. The healthcare workers working in primary care and wellness centres in remote areas may play a pivotal role in bridging the gap between patients with mental healthcare needs and mental health professionals. A parallel tele-networking involving the pharmacy may facilitate the delivery of medicines at doorstep following teleconsultation. Teleconsultation can facilitate multidisciplinary collaboration more easily than direct consultations in a time convenient manner. Use of digital educational materials and videos can pass on proper health education to a larger audience by reducing the task of treating clinician. Teleconsultation may be going to become more sensitive tool for assessment, diagnosis, communication, delivery of personalized care by using the concepts of artificial intelligence [9]. Researchers are using various algorithms to analyse data, which may help in accurate prediction related to diagnosis, selection of treatment as well as anticipating outcome.

Dr. Sujita Kumar Kar, Department of Psychiatry, King George's Medical University, Lucknow 226003, India.
Dr. Shailendra K Saxena, Centre for Advanced Research (CFAR), Faculty of Medicine; King George’s Medical University Lucknow 226003, India.
Dr. Russell Kabir, School of Allied Health, Faculty of Health, Education, Medicine, and Social Care, Anglia Ruskin University, Chelmsford, United Kingdom.

Dated the 28 October 2020

## References

[1] Agrawal A Bridging digital health divides. Science 2020; 369: 1050-1052. https://doi.org/10.1126/science.abc9295 10.1126/science.abc9295 PMid: 32855322

[2] Romanick-Schmiedl S Raghu G Telemedicine - maintaining quality during times of transition. Nature Reviews Disease Primers 2020; 6: 1-2. https://doi.org/10.1038/s41572-020-0185-x 10.1038/s41572-020-0185-x PMid: PMCid: 32483168 PMC7262488

[3] Sousa A Karia S Telepsychiatry during COVID-19: Some clinical, public health, and ethical dilemmas. Indian Journal of Public Health 2020; 64: 245-246. https://doi.org/10.4103/ijph.IJPH_511_20 10.4103/ijph.IJPH_511_20 PMid: 32496267

[4] Thara R Sujit J Mobile telepsychiatry in India. World Psychiatry 2013; 12: 84. https://doi.org/10.1002/wps.20025 10.1002/wps.20025 PMid: PMCid: 23471809 PMC3619179

[5] Gautham MS Gururaj G Varghese M The National Mental Health Survey of India (2016): Prevalence, socio-demographic correlates and treatment gap of mental morbidity. Int J Soc Psychiatry. 2020 6;66(4):361-372. https://doi:10.1177/0020764020907941 10.1177/0020764020907941 PMid: . 32126902

[6] Marcin JP Shaikh U Steinhorn RH Addressing health disparities in rural communities using telehealth. Pediatric Research 2016; 79: 169-176. https://doi.org/10.1038/pr.2015.192 10.1038/pr.2015.192 PMid: 26466080

[7] Ventriglio A Torales J Castaldelli-Maia J Telepsychiatry and social psychiatry. Int J Soc Psychiatry 2017; 63: 387-388. https://doi.org/10.1177/0020764017691552 10.1177/0020764017691552 PMid: 28701084

[8] Das S Manjunatha N Kumar CN Tele-psychiatric after care clinic for the continuity of care: A pilot study from an academic hospital. Asian Journal of Psychiatry 2020; 48: 101886. https://doi.org/10.1016/j.ajp.2019.101886 10.1016/j.ajp.2019.101886 PMid: 31835142

[9] Kuziemsky C Maeder AJ John O Role of Artificial Intelligence within the Telehealth Domain. Yearb Med Inform 2019; 28: 35-40. https://doi.org/10.1055/s-0039-1677897 10.1055/s-0039-1677897 PMid: PMCid: 31022750 PMC6697552

